# Generational perspectives and advocacy barriers among community health workers: implications for public health workforce leadership

**DOI:** 10.3389/fpubh.2025.1616506

**Published:** 2025-08-12

**Authors:** Venus Gines, Joanna Rodriguez, Diana Lobaina, Lea Sacca, Mariarebecca Torres, Maria C. Mejia

**Affiliations:** ^1^Dia de la Mujer Latina, Inc., Katy, TX, United States; ^2^Department of Family and Community Medicine, Baylor College of Medicine, Houston, TX, United States; ^3^Department of Population Health, Schmidt College of Medicine, Florida Atlantic University, Boca Raton, FL, United States

**Keywords:** community health workers, workforce development, public health leadership, generational differences, health equity, health policy

## Abstract

Community Health Workers (CHWs) play a crucial role in the public health workforce, particularly within underserved communities where they enhance access to healthcare, address social determinants of health, and advocate for health-related policy changes. This Perspective examines how age and experience influence CHWs’ involvement in workforce diversity initiatives, advocacy activities, and job satisfaction. Using data from a cross-sectional survey conducted among 306 CHWs during statewide workforce development events in Texas, United States, we identified significant generational and experiential differences in CHW engagement. Younger CHWs (aged 20–30 years) were significantly more inclined to prioritize mentorship and role modeling (*p* = 0.011), while older CHWs (aged 61 years and above) favored policy-driven advocacy (*p* = 0.003). Limited funding was the most frequently reported barrier (48.4%), and CHWs with less than 1 year of experience reported higher levels of organizational barriers and discrimination. Job satisfaction notably increased with age, reaching 73% among CHWs aged 61 years and older. These insights underscore the need for tailored strategies to strengthen workforce retention, leadership cultivation, and systemic support for CHWs, ultimately enhancing public health infrastructure.

## Introduction

Community Health Workers (CHWs) serve as a bridge between healthcare systems and the communities they represent, playing a key role in advancing health equity and improving outcomes in underserved populations (1, 2). Their lived experience and cultural alignment with the populations they serve make them uniquely suited to promote patient engagement, deliver education, and advocate for systemic change. As the CHW workforce grows nationally, attention has increasingly turned toward the importance of workforce development, retention, and leadership cultivation (3, 4).

During the COVID-19 pandemic, CHWs became lifelines for communities hardest hit by misinformation, isolation, and lack of access to healthcare (5). They translated complex public health guidance, delivered life-saving resources, and bridged trust where formal systems had failed (5). Historically, CHWs have evolved from informal volunteers to formally recognized healthcare team members, a progression recognized in both academic literature and federal health initiatives. Their expanded roles have significantly improved community-level health outcomes in underserved populations, with systematic reviews documenting reduced hospitalizations and improved chronic disease management (6). However, despite increased recognition in healthcare policy, CHWs continue to encounter persistent disparities in integration, utilization, and professional development. Variability in certification standards, reimbursement policies, and institutional support creates a disconnect between policy aspirations and real-world implementation, pointing to a pressing need for structural reforms.

Despite their value, CHWs frequently face systemic barriers, including insufficient funding, lack of institutional support, and workplace discrimination (7, 8). These barriers vary based on demographic and experiential factors, but little is known about how such dimensions influence CHWs’ engagement in advocacy, satisfaction, or leadership development (9).

In both high- and low-resource settings worldwide, CHWs have demonstrated effectiveness in improving chronic disease management, increasing preventive screening uptake, and reducing health disparities by acting as trusted intermediaries between health systems and marginalized populations (6, 10). Evidence from countries such as South Africa and Ethiopia highlights that intrinsic motivation, community embeddedness, and clear career pathways are essential to CHW satisfaction and long-term engagement (11, 12). However, even in countries with formal CHW programs, such as the United States (US), barriers to professional advancement remain. Many CHWs lack access to structured leadership development, supervisory support, and career mobility, limiting the full realization of their potential as frontline public health leaders (13). These challenges underscore the need for sustained investment in CHW workforce development that is responsive to both global evidence and local contexts.

### Survey context and methods

To better understand how generational and experiential factors influence CHW engagement and perceptions, we conducted a cross-sectional survey of 306 CHWs in the state of Texas, United States (US). Data were collected during two large statewide workforce development and advocacy events held in April and May 2023. These gatherings brought together CHWs, trainers, policy advocates, and institutional leaders with the aim of promoting community health leadership and systems change.

The 15-item survey instrument included both multiple-choice and open-ended questions. It captured self-reported data on CHW demographics, language preference, job satisfaction, diversity strategies, barriers to advocacy, and experiences of bias or discrimination. Participants self-identified their age group (20–30, 31–40, 41–50, 51–60, or 61+) and years of CHW experience (<1 year, 1–3, 3–5, 5–10, 10–20, or >20 years). Bivariate analyses were conducted using chi-square tests to assess associations between these independent variables and workforce outcomes. All statistical analyses were conducted using STATA/SE 17. Missing data were addressed through listwise deletion. The survey was approved by the Baylor College of Medicine Institutional Review Board. While the survey was exploratory in nature, it provides valuable insight into how CHWs at different career stages navigate structural challenges and opportunities for leadership.

### Generational and experiential trends in CHW engagement

Our survey revealed notable generational and experience-based differences in advocacy engagement, perceived barriers, and job satisfaction among CHWs. Younger CHWs (aged 20–30 years) were significantly more likely to prioritize mentorship and role modeling (*p* = 0.011), suggesting their preference for peer engagement and professional growth through interpersonal connections (Table 1). Conversely, CHWs aged 61 years and older strongly preferred policy-driven strategies (*p* = 0.003), indicating a strategic orientation toward systemic advocacy and health policy leadership as their careers progressed.

**Table 1:** Key findings by age group and years of experience (*N* = 306).

Variable	Preferred diversity strategy by age group							
Age	Serving as role models	*p*-value	Advocating for diverse representation	*p*-value	Providing mentorship	*p*-value	Other	*p*-value
20–30 years (*n* = 31)	27 (87%)	0.011*	30 (97%)	0.003*	24 (77%)	0.712	6 (19%)	0.333
31–40 years (*n* = 72)	39 (54%)		54 (75%)		47 (65%)		7 (10%)	
41–50 years (*n* = 59)	36 (61%)		52 (88%)		38 (64%)		3 (5%)	
51–60 years (*n* = 104)	56 (54%)		77 (74%)		73 (70%)		11 (11%)	
61+ years (*n* = 40)	27 (68%)		37 (93%)		28 (70%)		4 (10%)	

Variable	Overall satisfaction with role by age group				
Age	Very satisfied	Somewhat satisfied	Neutral	Somewhat dissatisfied	*p*-value
20–30 years (*n* = 31)	16 (52%)	8 (26%)	4 (13%)	2 (6%)	0.651
31–40 years (*n* = 72)	41 (57%)	15 (21%)	6 (8%)	3 (4%)	
41–50 years (*n* = 59)	38 (64%)	8 (14%)	10 (17%)	1 (2%)	
51–60 years (*n* = 104)	61 (59%)	19 (18%)	10 (10%)	3 (3%)	
61+ years (*n* = 40)	29 (73%)	5 (13%)	4 (10%)	0 (0%)	

Variable	Experienced discrimination			Experienced stereotyping		
Age	Yes	No	*p*-value	Yes	No	*p*-value
20–30 years (*n* = 31)	8 (26%)	23 (74%)	0.482	13 (42%)	18 (58%)	0.856
31–40 years (*n* = 72)	25 (35%)	47 (65%)		28 (39%)	44 (61%)	
41–50 years (*n* = 59)	18 (31%)	41 (69%)		24 (41%)	35 (59%)	
51–60 years (*n* = 104)	35 (34%)	69 (66%)		35 (34%)	69 (66%)	
61+ years (*n* = 40)	8 (20%)	32 (80%)		14 (35%)	26 (65%)	

Variable	Experienced barriers by age group			
Age	Yes	No	Unsure	*p*-value
20–30 years (*n* = 31)	20 (65%)	6 (19%)	5 (16%)	0.673
31–40 years (*n* = 72)	53 (74%)	13 (18%)	6 (8%)	
41–50 years (*n* = 59)	39 (66%)	15 (25%)	5 (8%)	
51–60 years (*n* = 104)	67 (64%)	24 (23%)	10 (10%)	
61+ years (*n* = 40)	28 (70%)	11 (28%)	1 (3%)	

Variable	Types of barriers by age group							
Age	Lack of support from healthcare organizations	*p*-value	Resistance from healthcare providers	*p*-value	Limited funding	*p*-value	Other	*p*-value
20–30 years (*n* = 31)	11 (35%)	0.237	5 (16%)	0.022*	13 (42%)	0.914	8 (26%)	0.272
31–40 years (*n* = 72)	26 (36%)		21 (29%)		34 (47%)		13 (18%)	
41–50 years (*n* = 59)	16 (27%)		9 (15%)		30 (51%)		9 (15%)	
51–60 years (*n* = 104)	40 (38%)		25 (24%)		50 (48%)		11 (11%)	
61+ years (*n* = 40)	20 (50%)		17 (43%)		21 (53%)		5 (13%)	

*Statistically significant.

Job satisfaction exhibited a positive association with age: 73% of CHWs aged 61+ reported being “very satisfied,” compared to 52% of younger CHWs (20–30 years). Additionally, younger CHWs reported higher rates of workplace bias and stereotyping (42%) than their older counterparts (34–35%). Though the difference in reported bias was not statistically significant, the data underscore a critical challenge potentially impacting retention among early-career CHWs (Table 1).

Similarly, years of experience influenced advocacy engagement and barriers. Providing mentorship emerged as the only diversity advocacy strategy significantly associated with experience, with seasoned CHWs (10 + years) more actively engaging in mentorship roles (*p* = 0.036, Table 2). Provider resistance was significantly more common among CHWs with over 20 years of experience (42%, *p* = 0.011), who also frequently reported funding limitations (74%) and inadequate organizational support (42%). Despite these barriers, highly experienced CHWs consistently expressed higher job satisfaction (68%), highlighting their professional resilience.

**Table 2:** Key findings by years of experience (*N* = 306).

Variable	Preferred diversity strategy by years of experience							
Years of experience	Serving as role models	*p*-value	Advocating for diverse representation	*p*-value	Providing mentorship	*p*-value	Other	*p*-value
<1 year (*n* = 77)	41 (53%)	0.414	59 (77%)	0.117	46 (60%)	0.027*	8 (10%)	0.942
1–3 years (*n* = 41)	24 (59%)		36 (88%)		32 (78%)		4 (10%)	
3–5 years (*n* = 46)	32 (70%)		41 (89%)		36 (78%)		9 (20%)	
5–10 years (*n* = 83)	51 (61%)		67 (81%)		58 (70%)		6 (8%)	
10–20 years (*n* = 39)	22 (56%)		28 (72%)		21 (54%)		3 (8%)	
>20 years (*n* = 19)	14 (74%)		18 (95%)		16 (84%)		1 (5%)	

Variable	Experienced barriers by years of experience			
Years of experience	Yes	No	Unsure	*p-*value
<1 year (*n* = 77)	51 (66%)	16 (21%)	10 (13%)	0.110
1–3 years (*n* = 41)	31 (76%)	10 (24%)	0 (0%)	
3–5 years (*n* = 46)	32 (70%)	9 (20%)	5 (10%)	
5–10 years (*n* = 83)	56 (67%)	16 (19%)	9 (14%)	
10–20 years (*n* = 39)	20 (51%)	15 (38%)	34 (11%)	
>20 years (*n* = 19)	16 (84%)	3 (16%)	0 (0%)	

Variable	Types of barriers by years of experience							
Years of experience	Lack of support from healthcare organizations	*p*-value	Resistance from healthcare providers	*p*-value	Limited funding	*p*-value	Other	*p*-value
<1 year (*n* = 77)	27 (35%)	0.759	9 (12%)	0.011*	33 (43%)	0.173	13 (17%)	0.839
1–3 years (*n* = 41)	15 (37%)		9 (22%)		23 (56%)		8 (20%)	
3–5 years (*n* = 46)	21 (46%)		12 (26%)		22 (48%)		8 (17%)	
5–10 years (*n* = 83)	29 (35%)		29 (35%)		39 (47%)		10 (12%)	
10–20 years (*n* = 39)	12 (31%)		9 (23%)		16 (41%)		5 (13%)	
>20 years (*n* = 19)	8 (42%)		8 (42%)		14 (74%)		2 (11%)	

*Statistically significant.

## Discussion

This Perspective emphasizes the importance of considering age and years of experience as critical factors in CHW workforce development. CHWs are increasingly recognized as key public health leaders, particularly within underserved communities, yet their ability to effectively advocate, lead, and sustain engagement is frequently influenced by access to mentorship, resources, and organizational support (13). Younger CHWs contribute valuable energy, innovation, and dedication to health equity initiatives, yet they disproportionately face discrimination, professional isolation, and insufficient institutional support (14). Conversely, veteran CHWs demonstrate significant resilience, job satisfaction, and a strong sense of professional purpose, even when confronted by ongoing systemic challenges such as limited funding, inconsistent institutional recognition, and resistance from healthcare providers (11, 12).

These generational and experiential differences align closely with international CHW workforce research findings. Studies conducted in Ethiopia and India report that younger health workers experience higher rates of burnout, lower levels of autonomy, and increased emotional stress (14, 15). Specifically, Ethiopian CHWs in early career stages frequently expressed lower satisfaction due to demanding workloads and inadequate supervisory support (15). On the other hand, experienced CHWs, despite navigating chronic resource shortages, often reported greater alignment with their professional purpose, driven largely by intrinsic motivation and a desire to mentor and support newer colleagues (16). Similar patterns have been identified in South African settings, highlighting intrinsic motivation, structured career pathways, and supportive supervision as key determinants of job satisfaction among CHWs (11).

These findings reinforce national discussions advocating policy reforms aimed at strengthening structural support for CHWs (13). Adopting a lifespan approach to CHW workforce development is essential, involving strategic investments in training, mentorship, sustainable funding mechanisms, and clearly defined leadership pathways tailored to each career stage. By systematically addressing these workforce gaps, public health leaders can strengthen CHWs’ capacity to advocate for community needs, foster organizational cultures of inclusion, and support meaningful systems change. International evidence from Ethiopia, India, and South Africa underscores that structured supervision, consistent remuneration, and ongoing professional recognition are critical determinants of CHW motivation, job satisfaction, and overall effectiveness (11–14).

To ensure sustainable public health infrastructure, it is essential that CHWs be positioned as co-leaders and strategic partners within healthcare delivery and policy advocacy rather than viewed solely as auxiliary staff. This strategic shift involves incorporating CHWs into decision-making processes, developing structured mentorship and training programs, and creating supportive environments to prevent burnout and attrition. Integrating CHWs into interdisciplinary healthcare teams, as demonstrated by successful models in community health centers managing chronic conditions, highlights the potential for CHWs to significantly improve health outcomes and reduce disparities when properly supported (6, 10).

Future public health initiatives should consider how intersectional factors, such as race, ethnicity, gender, and immigration status, intersect with age and experience to further influence CHWs’ professional trajectories and effectiveness. Addressing these complex dynamics requires systemic investments and targeted policy reforms. By drawing on domestic innovations and global best practices, the United States public health system can cultivate a CHW workforce that is resilient, well-supported, and reflective of the diverse communities it serves.

### Future directions for policy, practice, and education

The generational and experiential patterns identified among CHWs in this study highlight the urgent need for targeted and sustainable workforce development strategies. For early-career CHWs, robust mentorship programs can effectively bridge gaps in professional development, offering institutional support that is often lacking. These mentorship initiatives, integrated within community-based organizations and educational settings, can build confidence, reduce isolation, and foster peer learning. Given that younger CHWs disproportionately encounter workplace bias and report lower job satisfaction, structured mentorship and culturally responsive supervision become critical retention tools.

Experienced CHWs constitute a significant yet underutilized leadership resource. Their extensive knowledge of healthcare systems and advocacy expertise can significantly inform institutional reforms and community-based initiatives. Creating formal opportunities for veteran CHWs to assume leadership positions on advisory committees, task forces, and training development teams can optimize their impact.

Stable funding remains central to sustainable workforce strategies. Policymakers must prioritize long-term financing mechanisms to ensure CHWs and their supervisors receive consistent, fair compensation. Additionally, making CHW services reimbursable through Medicaid and other insurance providers is crucial for sustainability.

Programs aimed at supporting CHWs must be developed collaboratively with the CHWs themselves, ensuring cultural relevance and responsiveness to their lived experiences. Establishing trauma-informed supervision and structured feedback mechanisms will inform necessary system-level changes. Evidence from successful community-clinical collaborations demonstrates the significant potential for CHWs when roles are clearly defined and consistently funded (17, 18).

### Expanding CHW roles through policy and system-level investments

Historically, community health workers have evolved significantly from informal community volunteers to formally recognized, integral members of healthcare teams. The increasing acknowledgment of CHWs’ roles by healthcare policies has substantially improved community-level health outcomes, especially among underserved populations. Recent policy initiatives in the United States, such as the inclusion of CHWs in the Affordable Care Act (ACA, 2010) and dedicated workforce programs introduced by the Centers for Disease Control and Prevention and the Health Resources and Services Administration, represent significant advances in formalizing CHW roles (17–20). Despite these efforts, significant disparities remain in CHWs’ integration, professional recognition, and career pathways across different states.

Variability in state-level certification and reimbursement processes continues to create barriers to developing a sustainable and cohesive CHW workforce. Only a select number of states currently offer standardized certification pathways for CHWs, affecting career development, reimbursement eligibility, and overall professional recognition. Medicaid reimbursement policies for CHW-delivered services vary significantly among states, further complicating efforts to secure sustainable funding and consistent service delivery. Institutional support, structured career pathways, clear role definitions, and adequate compensation have emerged as critical factors influencing CHW job satisfaction and long-term retention (13). International evidence similarly highlights that structured supervision and consistent remuneration significantly enhance CHW satisfaction and motivation, resulting in improved community health outcomes (12, 14).

To fully leverage the potential of CHWs, targeted investments in leadership and advocacy training are needed. Such initiatives should actively involve CHWs in policy-making processes, elevating their lived experiences and insights in health systems planning. CHWs’ deep community connections uniquely position them to provide invaluable perspectives often overlooked by conventional healthcare professionals.

Integrating CHWs into interdisciplinary healthcare teams has demonstrated substantial success in improving patient outcomes and care coordination, particularly for chronic conditions such as diabetes and hypertension (6). Tailoring professional development strategies to different career stages can further enhance retention, motivation, and overall workforce effectiveness. Younger CHWs benefit from structured mentorship, peer-support networks, and comprehensive onboarding programs, while veteran CHWs can provide mentorship, policy expertise, and leadership in advocacy coalitions.

Ultimately, strategic and comprehensive investments in CHW workforce sustainability are vital for addressing increasingly complex public health challenges. Policies aimed at stabilizing funding, providing structured professional development, and formally recognizing CHW contributions within healthcare systems are essential to fostering a robust, equitable, and sustainable public health workforce.

## Conclusion

Community Health Workers are essential to advancing health equity and strengthening public health infrastructure. Recognizing and strategically addressing differences in their needs based on age and experience are critical steps toward building a more inclusive, resilient workforce. Immediate interventions such as structured mentorship programs, clearly defined career pathways, and sustainable funding mechanisms can significantly enhance CHWs’ effectiveness and job satisfaction. Long-term policy and system-level reforms should prioritize integrating CHWs as strategic co-leaders within healthcare teams and public health planning processes. By investing in CHWs across all stages of their professional lifespan, public health systems can better address complex health disparities, ultimately promoting lasting community wellness and equity.

## Data Availability

The raw data supporting the conclusions of this article will be made available by the authors, without undue reservation.
